# Computerized assessment of background parenchymal enhancement on breast dynamic contrast-enhanced-MRI including electronic lesion removal

**DOI:** 10.1117/1.JMI.11.3.034501

**Published:** 2024-05-02

**Authors:** Lindsay Douglas, Jordan Fuhrman, Qiyuan Hu, Alexandra Edwards, Deepa Sheth, Hiroyuki Abe, Maryellen Giger

**Affiliations:** University of Chicago, Department of Radiology Committee on Medical Physics, Chicago, Illinois, United States

**Keywords:** background parenchymal enhancement, breast MRI, fuzzy *c*-means, U-Net, machine learning

## Abstract

**Purpose:**

Current clinical assessment qualitatively describes background parenchymal enhancement (BPE) as minimal, mild, moderate, or marked based on the visually perceived volume and intensity of enhancement in normal fibroglandular breast tissue in dynamic contrast-enhanced (DCE)-MRI. Tumor enhancement may be included within the visual assessment of BPE, thus inflating BPE estimation due to angiogenesis within the tumor. Using a dataset of 426 MRIs, we developed an automated method to segment breasts, electronically remove lesions, and calculate scores to estimate BPE levels.

**Approach:**

A U-Net was trained for breast segmentation from DCE-MRI maximum intensity projection (MIP) images. Fuzzy c-means clustering was used to segment lesions; the lesion volume was removed prior to creating projections. U-Net outputs were applied to create projection images of both, affected, and unaffected breasts before and after lesion removal. BPE scores were calculated from various projection images, including MIPs or average intensity projections of first- or second postcontrast subtraction MRIs, to evaluate the effect of varying image parameters on automatic BPE assessment. Receiver operating characteristic analysis was performed to determine the predictive value of computed scores in BPE level classification tasks relative to radiologist ratings.

**Results:**

Statistically significant trends were found between radiologist BPE ratings and calculated BPE scores for all breast regions (Kendall correlation, p<0.001). Scores from all breast regions performed significantly better than guessing (p<0.025 from the z-test). Results failed to show a statistically significant difference in performance with and without lesion removal. BPE scores of the affected breast in the second postcontrast subtraction MIP after lesion removal performed statistically greater than random guessing across various viewing projections and DCE time points.

**Conclusions:**

Results demonstrate the potential for automatic BPE scoring to serve as a quantitative value for objective BPE level classification from breast DCE-MR without the influence of lesion enhancement.

## 1 Introduction

Background parenchymal enhancement (BPE) is a significant predictor of breast cancer risk, with greater BPE increasing the odds of developing cancer.^1^^–^^5^ BPE is qualitatively defined according to the Breast Imaging Reporting & Data System (BI-RADS^®^) as minimal, mild, moderate, or marked BPE based on the visually perceived volume and intensity of enhancement in normal breast fibroglandular tissue (FGT) after contrast injection for dynamic contrast-enhanced (DCE) MRI.^2^^,^^6^ The distribution of the enhancement through the breast over the course of a dynamic contrast series often occurs initially at the periphery of the FGT due to the pattern of blood inflow from the internal and lateral thoracic arteries, which then feeds into the retroareolar region, which is the last to enhance.^2^^,^^7^ Normal FGT tends to exhibit a slow early and persistent delayed uptake of contrast, although in some cases of moderate or marked BPE, there is a rapid early contrast uptake.^7^ Radiologists typically use visual assessment to rate BPE during the early phase images of the DCE series, around 1 to 2 min postcontrast. In many cases, tumor volumes can cause an overestimation of BPE by radiologists; the increased intensity of the tumor enhancement due to angiogenesis can inflate the visual assessment of BPE. Also in cases with marked BPE, it can become difficult to differentiate between tumor and normal FGT, thus reducing the sensitivity in breast cancer screening.^8^ These effects have contributed to the intraobserver variability in clinical BPE assessment that have been reported, thus necessitating an objective method for quantifying BPE.^4^

A number of groups have developed quantitative measures for BPE, but a general consensus of the most useful value has yet to be reached. Human-engineered and deep-learned features for BPE have been calculated from both single MRI slices and MRI volumes; some incorporate FGT or breast segmentations, whereas others rely on the entire image.^3^^,^^9^^–^^11^ Recently, one study that was based on a semiautomated segmentation algorithm achieved strong performance in distinguishing women who did and did not develop breast cancer using a quantitative BPE value.^11^ Additionally, another study found that the complexity of the BPE assessment caused only weak correlations between the investigators’ quantitative values and the associated clinical ratings.^12^ These studies demonstrate that further investigation is needed to develop a fully automated, objective method for quantifying BPE.

We have developed an automated machine learning method to segment breasts and electronically remove the influence of lesion presence on a computer BPE score.^13^ Our method was designed to mimic radiologist assessment of BPE from maximum intensity projections (MIPs), and it offers a robust estimation of BPE levels from breast DCE-MR projection images. We investigated the performance of computer BPE scores calculated from the second postcontrast subtraction MIPs of both breasts, the affected breast, and the contralateral, unaffected breast images created before and after the electronic removal of lesions. Additionally, we investigated the effect of various image parameters on the performance of computer BPE scores calculated from original and rescaled versions of maximum- or average-intensity projections (AIPs) of first- or second postcontrast subtraction DCE-MRI volumes.

## 2 Methods

### 2.1 Dataset

A dataset of 426 conventional breast DCE-MR exams (from 399 patients aged 23 to 89 years) was retrospectively collected at the University of Chicago over a span of 12 years (from 2005 to 2017) under HIPAA-compliant Institutional Review Board-approved protocols (Table 1). Routine bilateral breast MRIs were acquired using a Philips Achieva scanner with either 1.5 or 3 T magnet strength. The breast DCE-MRI protocol included a fat-saturated 3D T1 weighted spoiled gradient-echo sequence that was used to acquire pre- and postcontrast images with a temporal resolution of 60 to 75 s (TE = 2.2 to 2.8 ms, TR = 4.5 to 7.5 ms, flip angle = 10 deg to 20 deg, in-plane resolution = 0.5 to 1.0 mm, FOV = 28.0 to 44.1 cm, matrix=320−552×256−525, slice thickness = 1 to 3.5 mm, interslice gap = 0.8 to 2.5 mm). Radiologist BPE ratings were acquired from a prior clinical review. A subset of 76 exams from 73 patients (6 minimal, 18 mild, 26 moderate, 11 marked, and 15 unknown BPE) was set aside to use in developing the breast segmentation methods. A subset of the remaining exams, 350 exams (99 minimal, 159 mild, 78 moderate, and 14 marked BPE) from 326 patients, each with only one diagnosed lesion, was used for independent testing of the proposed machine learning algorithm for BPE. For each exam, the breast containing the diagnosed lesion is termed the “affected” breast, and the contralateral breast is termed the “unaffected” breast.

**Table 1:** Distribution of radiologist BPE ratings contained within the dataset of 426 DCE-MR exams from 399 patients.

	Minimal (no. of exams)	Mild (no. of exams)	Moderate (no. of exams)	Marked (no. of exams)	Unknown (no. of exams)	Total (no. of exams)
Training set	6	18	26	11	15	76
Test set	99	159	78	14	0	350
Total	105	177	104	25	15	426

All exams from a given patient were in either the training set or test set. Exams without a clinical BPE rating in the radiologist report were categorized as “unknown.”

### 2.2 Breast Segmentation

**Fig. 1:**
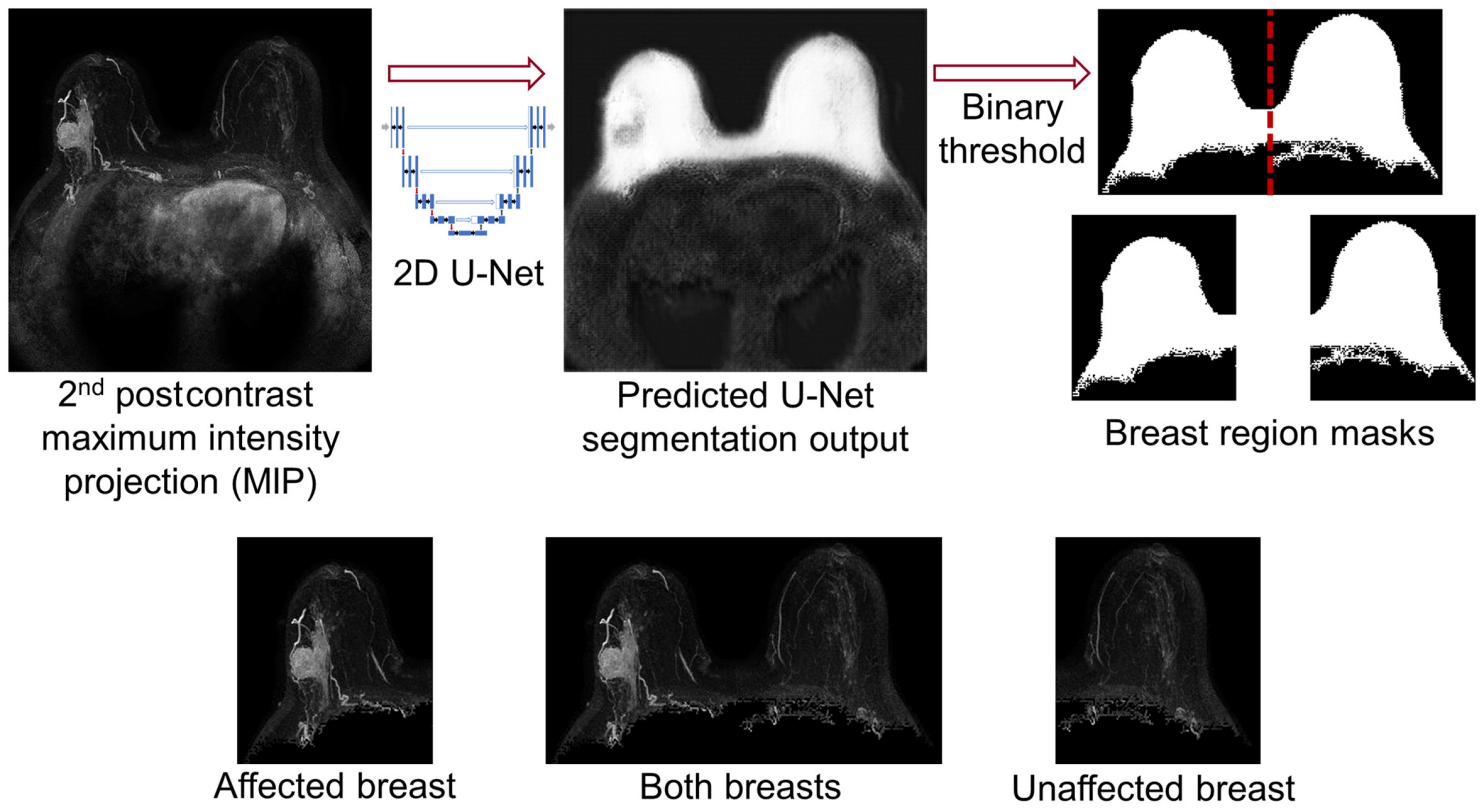
2D U-Net was trained for computerized breast segmentation on MIPs of the second postcontrast subtraction DCE-MRIs. A binary threshold was applied to the predicted U-Net output to generate breast region masks, and the individual breast regions were created by a vertical split at the center of the breast region containing both breasts.

The 2D U-Net convolutional neural network^14^ is capable of producing accurate segmentations when it is trained on a relatively small number of images;^15^ thus a training set of 76 exams was selected to contain a variety of lesion sizes and BPE levels represented in the full dataset. For the subset of 76 exams, an expert radiologist (7 years of experience in breast imaging) provided manual delineations of breast margins on the MIP of the second postcontrast subtraction image volume. The radiologist-delineated breast margins were used as the reference standard for training a U-Net for whole breast segmentation from second postcontrast subtraction MIPs, and visual assessment was used to qualitatively review the segmentation performance for the training set. The base U-Net model^14^ was trained using the Adam optimizer and a binary cross-entropy loss function; training was allowed to run for up to 200 epochs. The U-Net produced pixel probability map outputs with values ranging from 0 to 1, and a threshold of 0.25 was applied to convert the predicted U-Net outputs to binary segmentation images. To produce the breast region masks for use in our method, a subsequent postprocessing step was conducted to identify the largest object from the mask as the region containing both breasts. The region containing both breasts was vertically split at its center point to generate masks defining only the affected breast region and only the unaffected breast region. These breast masks were applied to the full postcontrast subtraction projection images to retain only the pixels belonging to both breasts, the affected breast, or the unaffected breast (Fig. 1). Without a radiologist reference available for the test cases, visual assessment was used to ensure that the binary mask sufficiently contained the entire breast region with minimal pixels from the chest wall.

### 2.3 Electronic Lesion Removal

**Fig. 2:**
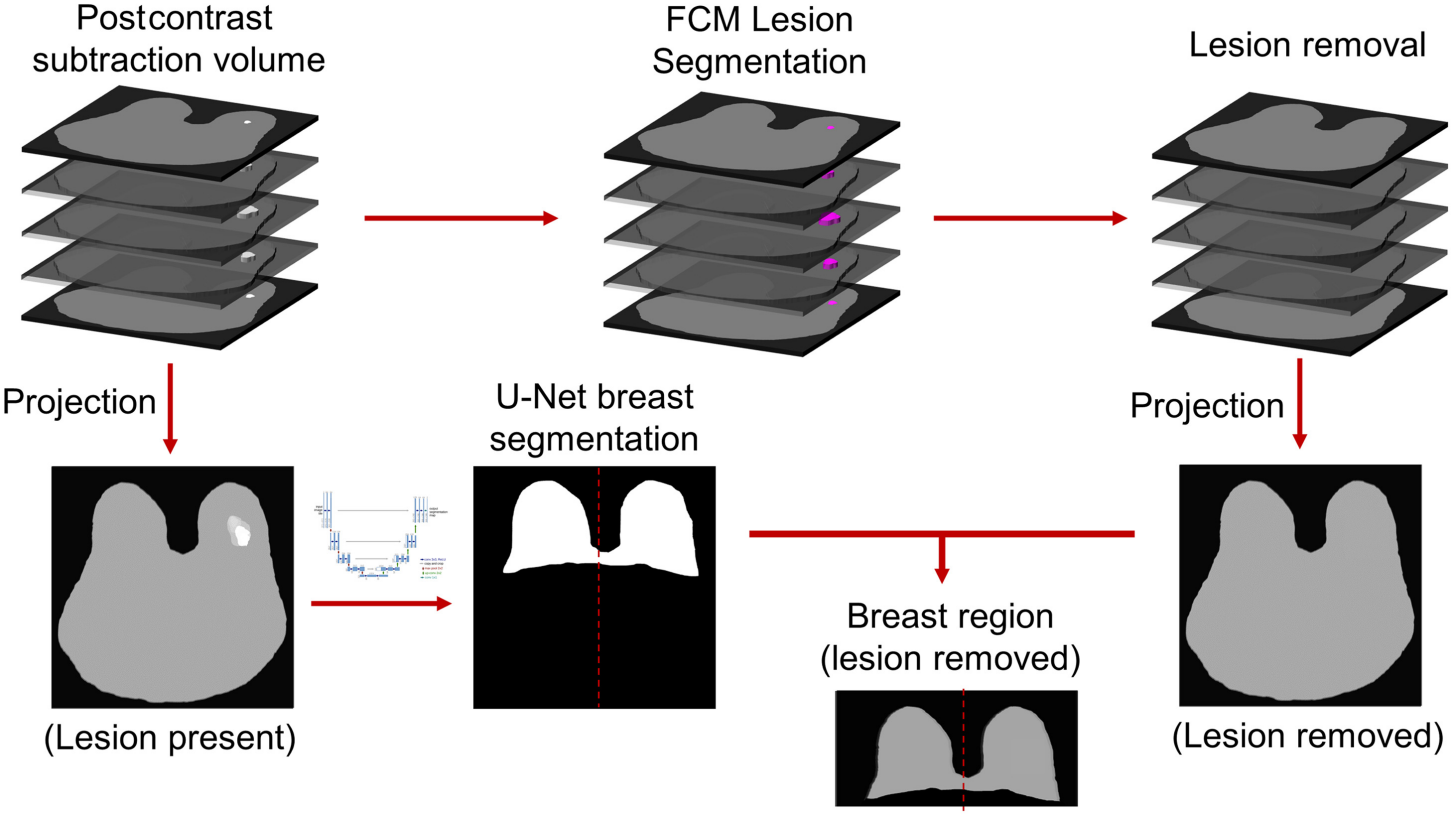
Flowchart of the method for electronic lesion removal, image projection, and breast segmentation from a postcontrast subtraction breast DCE-MRI. Lesion and breast segmentations were performed using FCM clustering and U-Net CNN, respectively. The breast mask was vertically split at the center to select the affected or unaffected breast regions from the projection image excluding the lesion. Computer BPE scores were calculated in a separate rescaled MIP after implementation of our digital electronic lesion removal algorithm.

A well-established, in-house, automated 3D fuzzy c-means (FCM) clustering approach was used to segment the lesions from the DCE-MR volumes.^16^ The lesion sizes, approximated by the square root of the lesion area at the center lesion slice, ranged between 2 and 65 mm. To electronically remove the lesions, the lesion area defined by the FCM segmentation was replaced with a value equivalent to the average intensity of the pixels bordering the lesion segmentation on the second postcontrast subtraction image slice. This process was repeated on each slice that passed through the lesion before projecting the maximum pixel values from all available volume slices to produce a new MIP that excluded the influence of the lesion. The breast masks generated from the U-Net outputs were used to retain only the pixels belonging to both breasts, the affected breast, and the unaffected breast on the second postcontrast subtraction MIP with the lesion removed (Fig. 2). For comparison across input image parameters, this method was also conducted using first postcontrast subtraction images and using average-intensity projections to produce images of the affected breast without the influence of the lesion.

### 2.4 Computer BPE Score

**Fig. 3:**
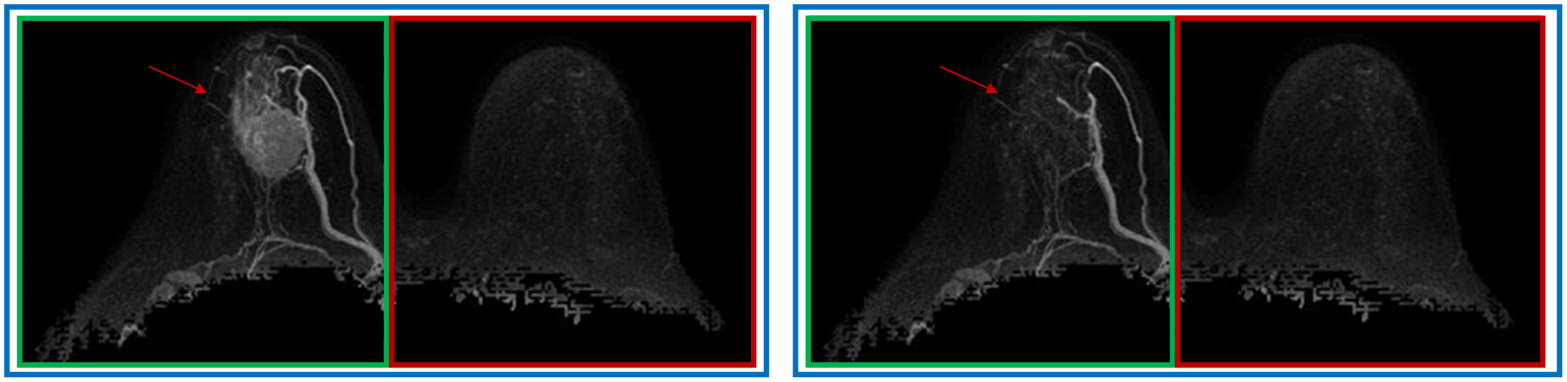
Computer BPE scores were calculated from the affected breast (green box), unaffected breast (red box), and both breasts (blue box), before (left) and after (right) lesion (red arrow) removal.

For each of the defined breast regions (both, affected, and unaffected), the computer BPE scores were automatically calculated from the second postcontrast subtraction MIPs. Within each MIP, the pixel values were rescaled so that the original pixel values ranging from 0 to 255 were scaled to a range of 0 to 1. To reflect the qualitative definitions of BPE assigned by radiologists based on the amount and intensity of the enhancement in FGT, the average pixel intensity of the pixels contained within each breast served as the computer BPE score (Fig. 3).

### 2.5 Evaluation of BPE Scores

**Fig. 4:**
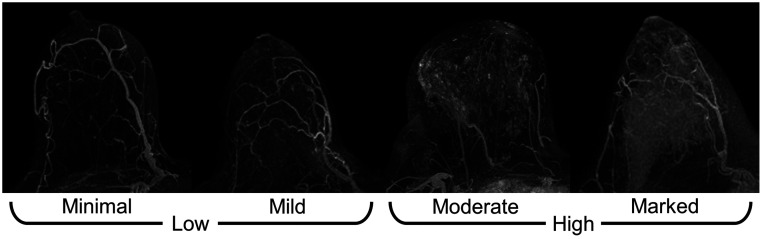
Clinical radiologist BPE ratings were used as the reference standard for ROC analysis. ROC analysis was performed to determine the predictive value of computer BPE scores for binary classification of minimal versus marked BPE and of low (minimal, mild) versus high (moderate, marked) BPE.

To determine the strength and direction of the correlation of the computer BPE scores with radiologist BPE ratings, Kendall’s tau-b was used in rank correlation with a t-test to determine the statistical significance of the correlation.^17^ To assess how lesion removal changes the computer BPE scores, the ratio of the computer BPE score calculated after lesion removal to the computer BPE score calculated before lesion removal was examined according to the lesion size for the second postcontrast subtraction MIP of each affected breast. Receiver operating characteristic (ROC) analysis was performed using the proper binormal model.^18^ Clinical radiologist BPE ratings were the only truth available for BPE assessment, so the performance of the computer BPE scores was compared with random guessing. To determine the predictive value of the computer-extracted BPE scores, ROC analysis was performed using computer BPE scores for binary classification of minimal versus marked BPE; it was also evaluated for binary classification of low (mild, minimal) versus high (marked, moderate) BPE (Fig. 4). The statistical significance of the area under the ROC curve (AUC) relative to random guessing was determined using the z-test with Bonferroni corrections for multiple comparisons.^19^

**Fig. 5:**
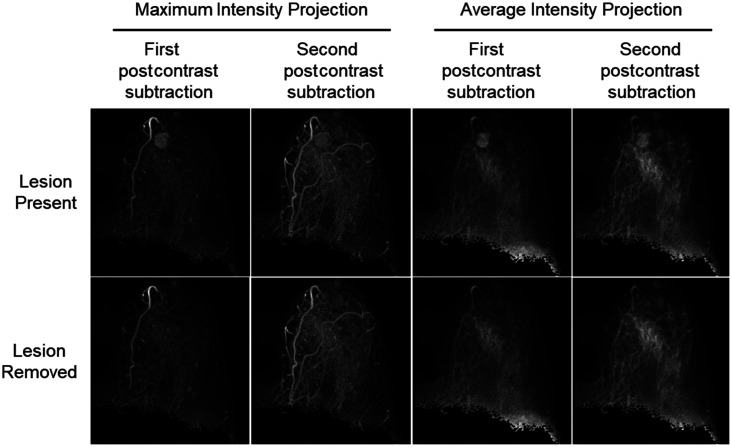
Example images of an affected breast from a case classified as marked BPE by a radiologist. The computer BPE scores were calculated from the affected breast region in the first- or second-postcontrast subtraction maximum- or average-intensity projection (MIP or AIP) images after electronic lesion removal (bottom row).

Rank correlation and ROC analysis were also used to understand the effect of different image parameters on the calculated BPE. The minimal versus marked BPE and low versus high BPE tasks were thus evaluated for computer BPE scores calculated from the affected breast in each of the image types (shown in Fig. 5).

## 3 Results

**Fig. 6:**
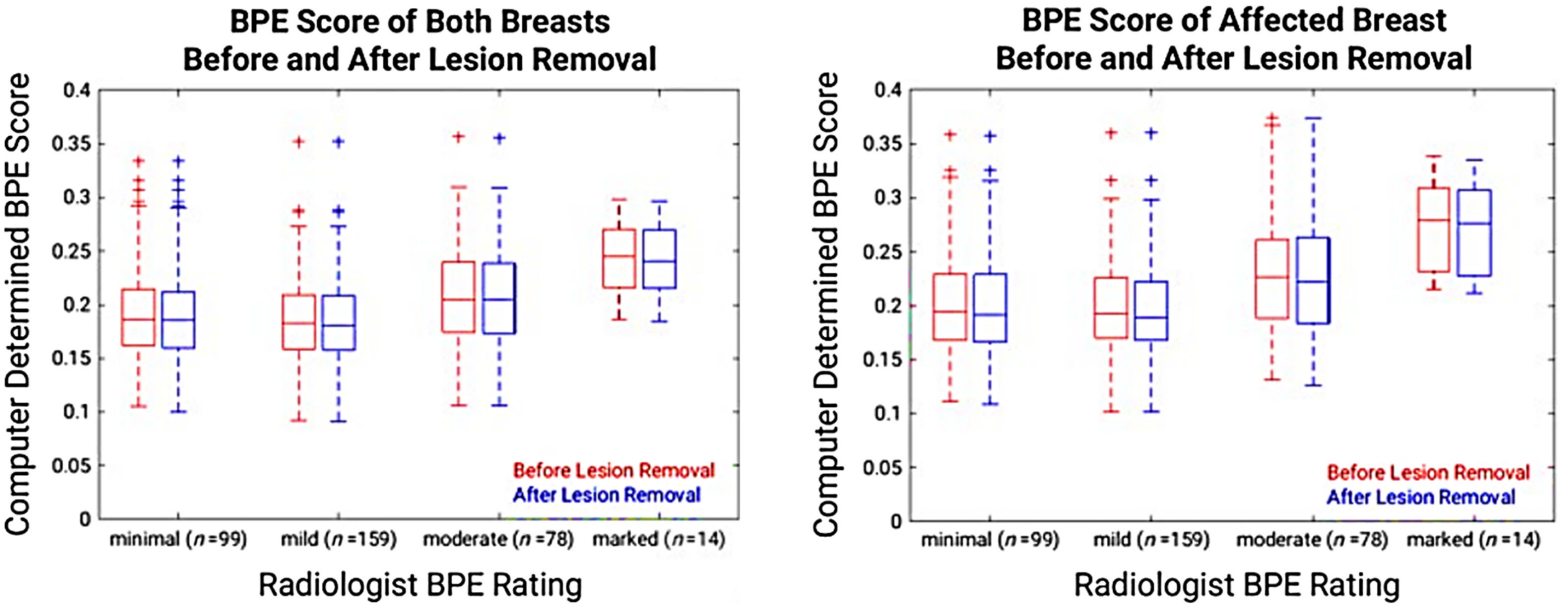
Positive correlation between all computer BPE scores (second postcontrast subtraction MIP) and the radiologist BPE ratings were statistically significant (p<0.001). BPE scores from unaffected breasts are not shown because there is no change in score after lesion removal.

On the independent set of 350 second postcontrast subtraction MIPs, a statistically significant positive correlation was found between the computer BPE scores and the radiologist BPE ratings for all breast regions, before and after the lesion removal (p<0.001, t-test) (Fig. 6).

**Fig. 7:**
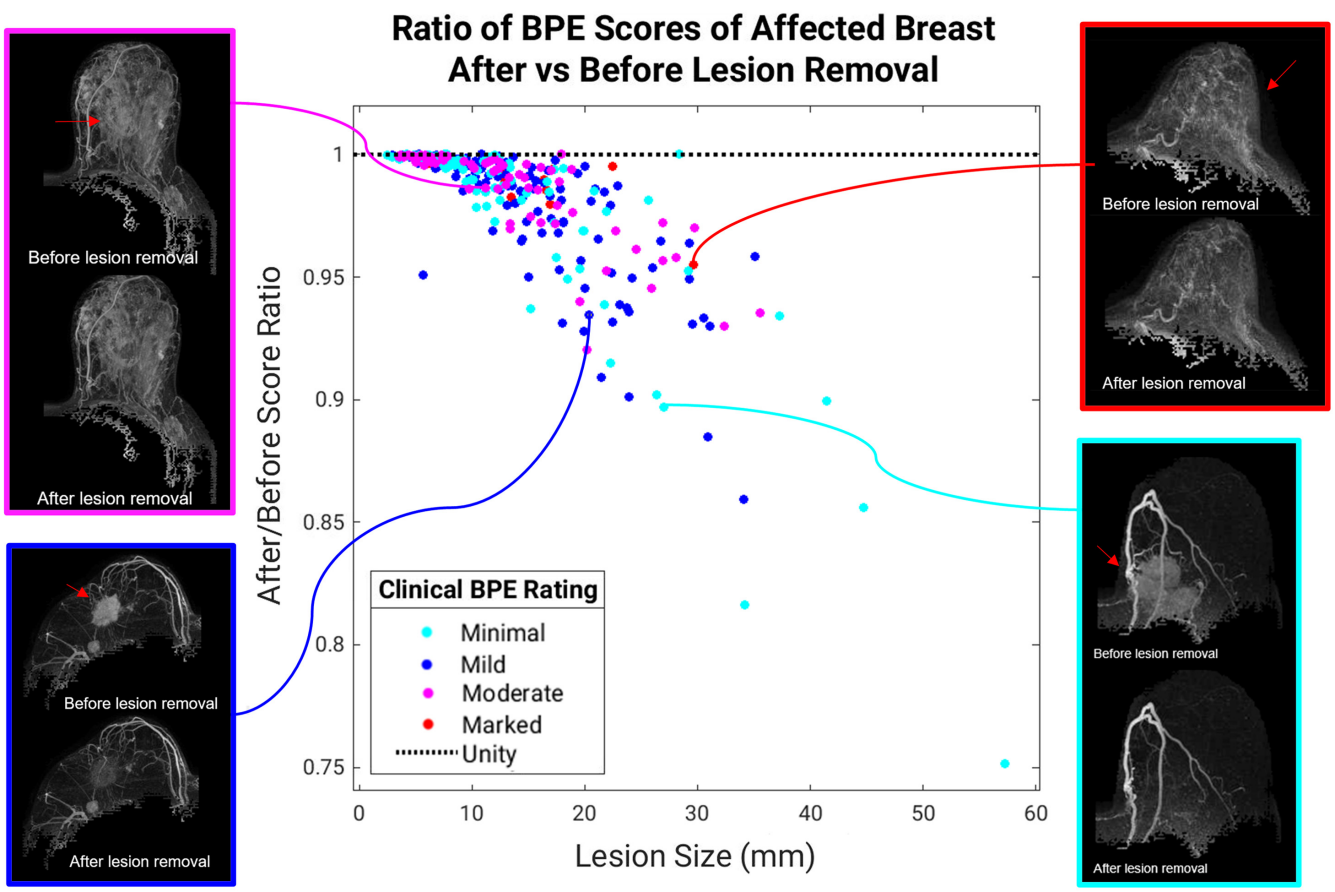
Ratio of the computer BPE score (second postcontrast subtraction MIP) calculated after lesion removal to the score calculated before lesion removal for the affected breast is shown versus the lesion size (n=350). Results demonstrate the importance of lesion removal to avoid inflation of computer BPE estimations, especially in cases containing large lesions and low BPE levels.

The ratio of the scores calculated after versus before lesion removal, sorted by size and BPE level, are shown with example cases of affected breasts (Fig. 7). As would be expected, the computer BPE scores were reduced after the lesion removal; this was more pronounced for larger lesions and cases with low BPE levels. More specifically, among the cases with lesions larger than 10 mm, the average computer BPE score was reduced by 3.83% for minimal BPE ratings and 1.48% for marked BPE ratings.

The AUCs in the task of classifying minimal versus marked BPE and in the task of classifying low versus high BPE according to radiologist ratings were calculated for each of the breast regions in the second postcontrast subtraction MIPs (Table 2). All classification tasks performed statistically significantly better than guessing (z-test). For all breast regions, the computer BPE scores yielded greater AUC results for minimal versus marked BPE than for low versus high BPE levels, which was expected because it is easier to distinguish between the two extreme BPE levels than the intermediate ones. The computer BPE scores from the affected breast, both before and after lesion removal, yielded greater AUC results than the computer BPE scores from the unaffected breast for both classification tasks; thus the computer BPE scores calculated from that region were used in subsequent evaluations.

**Table 2:** Effect of breast region used for computer BPE score.

	Minimal (n=99) versus marked (n=14) BPE AUC	Low (n=258) versus high (n=92) BPE AUC
Both breasts	0.84 ± 0.04 (p<0.0001)*	0.66 ± 0.03 (p<0.0001)*
Both breasts, removed lesion	0.83 ± 0.04 (p<0.0001)*	0.66 ± 0.03 (p<0.0001)*
Affected breast	0.86 ± 0.03 (p<0.0001)*	0.68 ± 0.03 (p<0.0001)*
Affected breast, removed lesion	**0.87 ± 0.04** (p<0.0001)*	**0.68 ± 0.03** (p<0.0001)*
Unaffected breast	0.79 ± 0.05 (p<0.0001)*	0.66 ± 0.03 (p<0.0001)*

AUC results from ROC analysis for the task of BPE level classification using computer BPE scores calculated from the rescaled second postcontrast subtraction MIP. High BPE includes marked or moderate BPE, and low BPE includes mild or minimal BPE. Raw, uncorrected p-vales from the z-test are reported in this table; however, statistical significance of the AUCs was assessed using the Bonferroni correction for 13 comparisons. Asterisks indicate performance statistically significantly greater than random guessing. The bolded selection was used in subsequent analyses.

**Fig. 8:**
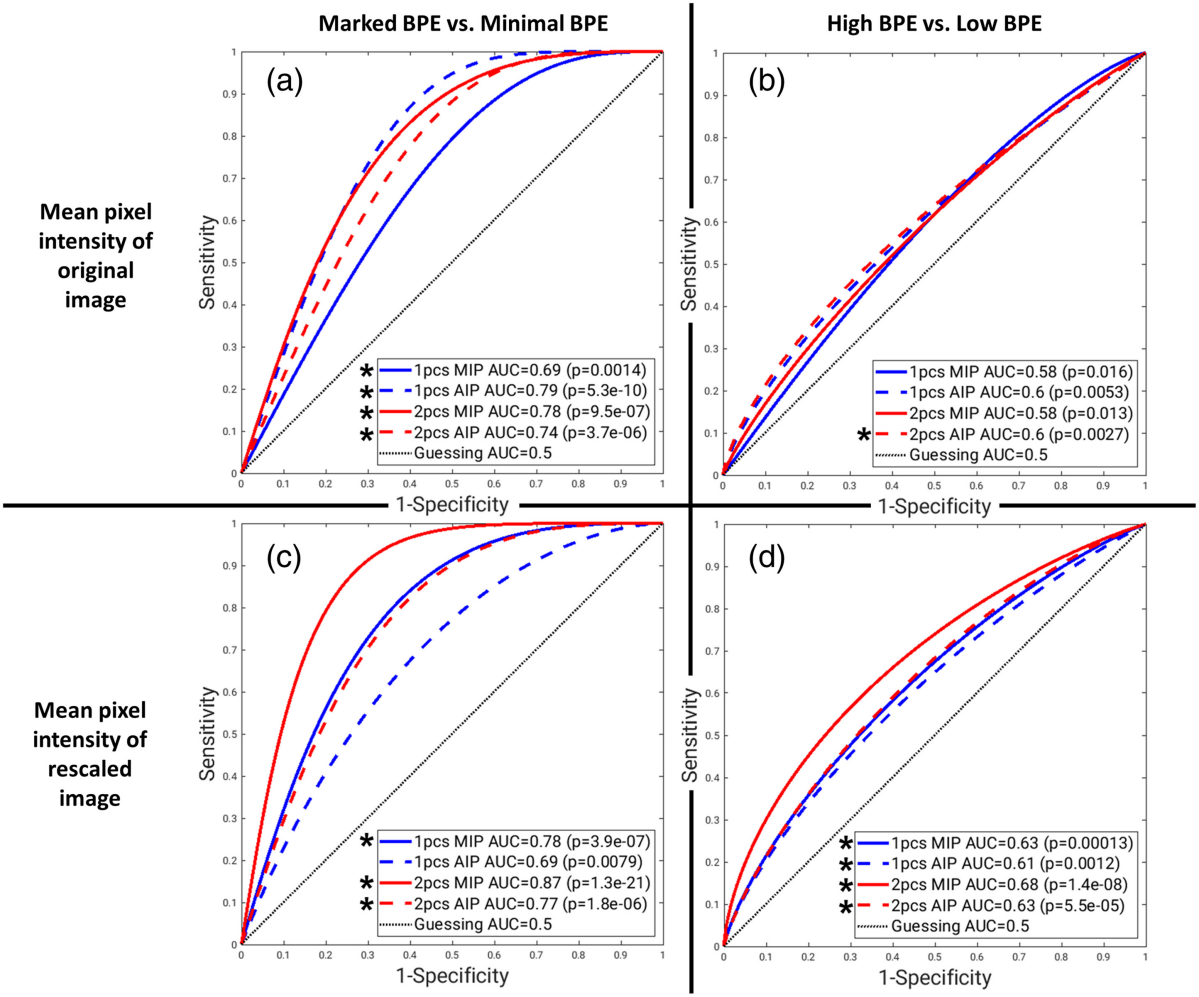
ROC curves for the binary classification tasks of marked BPE (n=14) versus minimal BPE (n=99) (a), (c) and high (marked or moderate) BPE (n=92) versus low (mild or minimal) BPE (n=258) (b), (d) using the mean pixel intensity of (a), (b) the original and (c), (d) rescaled image of the affected breast. Asterisks indicate classification performance statistically significantly greater than random guessing. Raw, uncorrected p values are reported from the z-test; statistical significance for performance greater than random guessing was assessed after a Bonferroni correction for 13 comparisons. 1pcs, 2pcs: first- and second-postcontrast subtractions; MIP, AIP: maximum- and average-intensity projection.

The results of the comparisons between computer BPE scores calculated from varying image types are shown in Table 3 and Fig. 8 (affected breast scores only). Statistically significant correlations were found between the radiologist BPE ratings and the computer extracted BPE scores from the rescaled images, except for the first postcontrast subtraction AIP. Computer BPE scores performed statistically significantly greater than random guessing in minimal versus marked BPE level classification, except for the first postcontrast subtraction AIP. Computer BPE scores performed statistically significantly greater than random guessing in low versus high BPE level classification, except for the mean of original MIPs and original first postcontrast subtraction AIP. For all image types, the computer BPE scores yielded greater AUC results for minimal versus marked BPE than for low versus high BPE levels. In both BPE level classification tasks, computer BPE scores from rescaled images yielded greater AUC results than from original images. ROC curves showed that computer BPEs from second postcontrast-projections yielded greater AUC results than first postcontrast-projections and computer BPE scores from MIPs yielded greater AUC results than computer BPE scores from AIPs. Compared with the other image types, the computer BPE scores of the rescaled second postcontrast MIP statistically significantly outperformed other rescaled image types for minimal versus marked BPE classification (p<0.05, z-test).

**Table 3:** Effect of breast image parameters used for the computer BPE score.

Quantitative value	Projection image type	Postcontrast subtraction time point	Kendall’s rank correlation tau-b (n=350)	Minimal (n=99) versus marked (n=14) BPE AUC	Low (n=258) versus high (n=92) BPE AUC
Mean pixel intensity of original image	Maximum	First	0.043 (p=0.299)	0.69 ± 0.06 (p<0.01)*	0.58 ± 0.03 (p=0.016)
		Second	0.075 (p=0.067)	0.78 ± 0.06 (p<0.0001)*	0.58 ± 0.03 (p=0.013)
	Average	First	0.083 (p=0.043)	0.79 ± 0.05 (p<0.0001)*	0.60 ± 0.03 (p<0.01)
		Second	0.090 (p=0.030)	0.74 ± 0.05 (p<0.0001)*	0.60 ± 0.03 (p<0.01)*
Mean pixel intensity of rescaled image	Maximum	First	0.132 (p<0.01)*	0.78 ± 0.06 (p<0.0001)*	0.63 ± 0.03 (p<0.001)*
		Second	0.186 (p<0.0001)*	0.87 ± 0.04 (p<0.0001)*	0.68 ± 0.03 (p<0.0001)*
	Average	First	0.119 (p<0.01)	0.69 ± 0.07 (p<0.01)	0.61 ± 0.03 (p<0.01)*
		Second	0.160 (p<0.0001)*	0.77 ± 0.06 (p<0.0001)*	0.63 ± 0.03 (p<0.0001)*

Results from Kendall’s rank correlation and ROC analysis for computer BPE scores calculated from the affected breast region. High BPE includes marked or moderate BPE, and low BPE includes mild or minimal BPE. Raw, uncorrected p-vales from the t-test or z-test are reported in this table; however, statistical significance of the AUCs was assessed using the Bonferroni correction for thirteen comparisons. Asterisks on tau-b values indicate a statistically significant correlation between the computer BPE scores and radiologist BPE rating. Asterisks on AUC values are statistically significantly greater than random guessing.

## 4 Discussion

In current clinical settings, radiologist BPE ratings are subjectively assigned based on the relative volume and intensity of enhancement in normal fibroglandular breast tissue after contrast injection for DCE-MRI. This study presented an automated computer algorithm for the assessment of BPE and investigated the effect of using various breast DCE-MR image types. The results of this work demonstrate the promising performance of an automatic BPE scoring method, which yields computer BPE scores in classifying marked versus minimal BPE across various image viewing projections and DCE time points. Our method of computing BPE scores from breast DCE-MR MIP images was not influenced by the contrast enhancement within lesions, which currently causes intraobserver variability in clinical BPE level assessment, because the algorithm includes an electronic removal of the lesion.

The automatically calculated computer BPE scores from all breast regions had a statistically significant correlation with the radiologist BPE ratings, with the exception of one image type; thus the computer BPE scores had a positive correlation with increasing BPE. The ratio of the computer BPE scores calculated after lesion removal to before lesion removal demonstrate the importance of electronic lesion removal to avoid inflation of BPE estimations, especially in cases containing large lesions and low BPE levels. Although the computer BPE scores from the second postcontrast subtraction MIPs of the affected and unaffected breasts appeared similar in boxplots, computer BPE scores of the affected breast yielded greater AUC results than those of the unaffected breast in the prediction of radiologist BPE ratings. Based on the computer BPE scores from all breast regions, the classification of minimal versus marked BPE yielded greater AUC results than the classification of low versus high BPE, which was expected because it is easier to distinguish between the two extreme BPE levels than the intermediate ones.

Although we observed that the AUC in the task of BPE level classification increased from before to after lesion removal, we failed to show that it was a statistically significant increase. The electronic removal of the lesion from the affected breast increased AUC results in the predictions for the minimal versus marked task, but not for the low versus high task; this may be due to the complexity of the BPE levels considered in each task. Given that the removal of lesions had the greatest impact on reducing the computer BPE score for minimal BPE cases, the lesion removal would improve the classification of minimal versus marked BPE. In the low versus high task, however, the large prevalence of mild and moderate BPE cases contributes to the difficulty of the task due to the similarity between intermediate BPE levels that exists even after lesion removal. Additionally, the AUC results for computer BPE scores calculated from various image projections and postcontrast subtraction time points demonstrated the flexibility of the algorithm in BPE level classification tasks. Comparisons between the original and rescaled versions of the maximum- and average-intensity projections (MIP and AIP) created from the first or second postcontrast subtraction images of the affected breast demonstrated that the computer BPE scores calculated from the rescaled, second postcontrast subtraction MIP yielded the greatest overall AUC results. Therefore, of the scores evaluated in this study, the best computer-generated representations of the relative intensity and volume of enhancement qualitatively assessed by radiologists were the computer BPE scores of the rescaled, second postcontrast subtraction MIP.

Future investigations should be done to address the limitations of our study to improve the performance of computer BPE scores. For instance, although our method includes three-dimensional lesion segmentation, our BPE scoring method is limited to two-dimensional MIPs. Also the performance of the breast segmentation was limited to a qualitative visual assessment; thus there is potential to improve the breast segmentation process. In the future, including a quantitative analysis of the breast segmentation would facilitate an assessment of the variability in computer BPE scores based on the precision of the masks that define breast regions. Additionally, in this work, the computer BPE scores were calculated from MIPs that often contained major vasculature, which contain bright pixels that may inflate the computer estimation of BPE (a current limitation). Future investigations should aim to remove the influence of the vasculature’s enhancement, as we have already considered for lesions, to produce a more accurate representation of the FGT enhancement. The only truth that we had available to assess the performance of our computer BPE scores for BPE level classification tasks were the radiologist BPE ratings assigned during initial clinical review; thus our ROC analyses were limited to comparisons against random guessing performance. Further investigation of variability in the reference standards used for algorithm development may improve the overall performance of our method in BPE classification tasks. In addition, future investigations should determine the significance of the influence of lesion enhancement on radiologist BPE ratings. Allowing radiologists to reassess images after electronic lesion removal would provide the opportunity to perform more comprehensive analyses of the computer BPE scores as well.

Ongoing investigations of our machine learning method for BPE scoring are being performed using an independent dataset of high-risk screening patients to evaluate the role of computer BPE scores in breast cancer risk assessment. Similar to the approach of many artificial intelligence methods that use tumor features as prognostic markers, other image-based biomarkers, such as BPE, may be factored into clinical risk assessment models. Ultimately, we believe that computer BPE scores have the potential to improve the predictive value of breast cancer risk assessment models in the future.

## Data Availability

The data and code used for this manuscript, including the DCE-MRIs, ROIs, and the algorithm to assess BPE, are not publicly available due to patient privacy and data sharing agreements.
